# Comparing Enhanced Sampling Methods in Exploring the Conformational Space of β‑Catenin_17–48_

**DOI:** 10.1021/acs.jpcb.6c02031

**Published:** 2026-06-24

**Authors:** Laura I. Gil Pineda, Marcelo D. Polêto, Haley M. Michel, Ashley M. Goodberlet, Justin A. Lemkul

**Affiliations:** † Department of Biochemistry, Virginia Tech, Blacksburg, Virginia 24061, United States; ‡ Department of Biochemistry, Center for Drug Discovery, Virginia Tech, Blacksburg, Virginia 24061, United States

## Abstract

Intrinsically disordered proteins (IDPs) play critical roles in cellular signaling and regulation, yet their dynamic conformational landscapes make them difficult to characterize experimentally and computationally. Phosphorylation, one of the most common post-translational modifications, frequently occurs within intrinsically disordered regions and can modulate protein structure and function. Enhanced sampling molecular dynamics methods offer a potential route to more efficiently explore the diverse conformations accessible to IDPs, but systematic comparisons of their performance sampling the conformational landscape of such systems remain limited. Here, we evaluate the conformational sampling of the intrinsically disordered β-catenin_17–48_ peptide in both its nonphosphorylated and phosphorylated states using three enhanced sampling approaches: Gaussian-accelerated molecular dynamics (GaMD), metadynamics (METAD), and weighted ensemble simulations (WESTPA), in comparison with conventional molecular dynamics simulations. Two collective variables (CVs) were explored to guide sampling: the ϕ dihedral angles of the phosphorylation sites Ser33 and Ser37 and the end-to-end distance of the peptide. We found that different enhanced sampling methods explored distinct regions of conformational space rather than converging to a single ensemble, with GaMD largely overlapping with unbiased simulations. Notably, METAD and WESTPA more readily accessed conformational regions not observed in unbiased simulations. Analysis of the combined conformational ensembles identified intermediate conformations connecting the nonphosphorylated and phosphorylated states, which are preferentially sampled in simulations employing adaptive strategies. Additionally, the Ser33/Ser37 ϕ angle CV more effectively captures phosphorylation-dependent conformational shifts than the end-to-end distance metric. Together, these results highlight how both the choice of enhanced sampling strategy and the selection of collective variables influence the exploration of IDP conformational landscapes.

## Graphical abstract

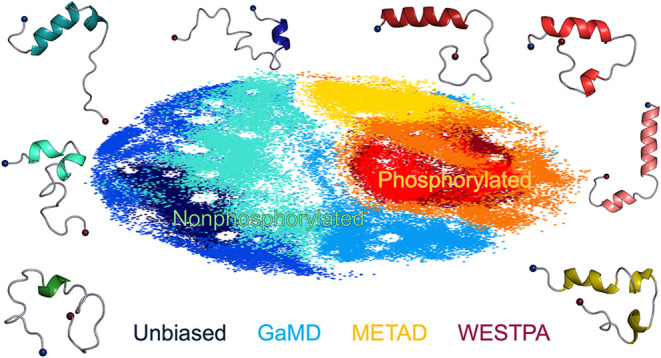

## Introduction

Intrinsically disordered proteins (IDPs) or intrinsically disordered regions are proteins or sequences enriched in polar and charged amino acids that lack a single, well-defined tertiary structure and instead exist as an ensemble of interconverting conformations.^1^ The wide range of conformations that IDPs can adopt can rapidly shift in response to environmental factors, post-translational modifications (PTMs), and binding events.^2^ This structural plasticity enables IDPs to play central roles in cellular processes such as signaling, regulation, molecular recognition, and the assembly of supramolecular complexes.^3^ However, disruption of IDP function through mutations or dysregulation of PTMs has been linked to numerous human diseases, including cancer,^4,5^ neurodegenerative disorders,^6,7^ and cardiovascular disease.^8^ For example, hyperphosphorylation of the microtubule-associated protein tau is strongly associated with the development of Alzheimer’s disease.^9^ Understanding how the conformational ensembles of IDPs govern their functional roles, and how these ensembles are altered under disease-associated conditions, is therefore critical for elucidating their mechanisms of action and their involvement in human health and disease.

Experimental methods such as NMR spectroscopy,^10,11^ single-molecule fluorescence Förster resonance energy transfer (smFRET),^12,13^ and small-angle X-ray scattering (SAXS)^14,15^ have been widely used to characterize the conformational ensembles of IDPs. However, these approaches primarily report on ensemble-averaged properties, with only smFRET providing access to subpopulations and dynamic behavior at the single-molecule level.^16^ Computational approaches, particularly molecular dynamics (MD) simulations, can complement these experiments by generating atomistic conformational ensembles and providing detailed structural insights.^16^ Nonetheless, computational methods come with their own challenges, including efficiently sampling the vast and complex conformational landscapes of IDPs. To address this limitation, there are a range of enhanced sampling techniques with different approaches^17^ like using biasing potentials, such as in Gaussian-accelerated molecular dynamics (GaMD),^18^ or adaptive sampling strategies, such as in weighted ensemble (WE) simulations.^19^ While these methods show promise for improving exploration of IDP conformational space, their relative performance and ability to capture functionally relevant conformations, particularly in the context of post-translational modifications, remain incompletely understood.

In this work, we used a peptide derived from the N-terminal intrinsically disordered region of β-catenin (residues 17–48) as a model system to evaluate how different enhanced sampling strategies capture phosphorylation-dependent conformational changes in IDPs. β-catenin_17–48_ is particularly well suited for this purpose, as phosphorylation at Ser33 and Ser37 has been shown experimentally to induce a transition from a largely disordered ensemble to one containing an N-terminal α-helical segment,^20^ providing a direct structural benchmark for comparison. The phosphorylation state of this region is also linked to β-catenin localization and activity,^21^ and mutations at these sites are associated with multiple cancers.^4^ To systematically assess sampling behavior, we compared GaMD,^22^ metadynamics (METAD),^23^ and WE simulations^24^ against conventional molecular dynamics simulations. By analyzing the conformational ensembles generated by each method, we aimed to determine how different sampling strategies influence exploration of the conformational landscape, the identification of intermediate or transition-like states, and the ability to capture experimentally observed phosphorylation-induced structural shifts. Agreement with experimental NMR observables, including scalar couplings and chemical shifts, was used to further evaluate the accuracy of the resulting ensembles. Together, this approach provides insights into which enhanced sampling strategies may be most appropriate for IDPs and how phosphorylation reshapes the conformational landscape of β-catenin.

## Methods

### System Setup and Unbiased MD Protocol

The nonphosphorylated and phosphorylated β-catenin_17–48_ structures (with Ser33 and Ser37 phosphorylated in the latter case) were obtained from the NMR study that investigated the effects of phosphorylation on β-catenin_17–48_ ^20^ and were provided directly by the authors (S. Megy, personal communication). The protein topologies were generated using the additive CHARMM36m (C36m) protein force field (FF)^25^ with the structures capped through N-terminal acetylation and C-terminal amidation to match experimental conditions. Each system was solvated in a cubic box of CHARMM-modified TIP3P water.^26–28^ The nonphosphorylated system had a net charge of zero and therefore required no additional ions, whereas neutralizing K^+^ counterions were added to the phosphorylated system. Next, the solvated system was relaxed via energy minimization in CHARMM^29^ via 500 steps of steepest descent minimization followed by 500 steps of adopted-basis Newton–Raphson minimization. Equilibration was performed under an *NPT* ensemble for 1 ns using OpenMM.^30^ During equilibration, position restraints (500 kJ/mol nm^2^) were applied to all non-hydrogen peptide atoms while water and K^+^ were free to diffuse. The Langevin integrator was used with a 2 fs integration step and a friction coefficient of 1 ps^–1^ to maintain temperature at 278 K, the experimental temperature at which the NMR spectra of the structures were collected. To maintain the pressure at 1 bar a Monte Carlo barostat was applied. Short-range van der Waals forces were switched smoothly to zero from 10 to 12 Å and electrostatic interactions were calculated via the particle mesh Ewald (PME) method^31,32^ with a real-space cutoff of 12 Å. The water molecules were held rigid via the SETTLE algorithm^33^ and all bonds to hydrogen were constrained using the SHAKE algorithm,^34^ allowing for the integration time step of 2 fs.

Next, Drude-2019 polarizable FF (Drude FF)^35^ topologies were generated for both systems by using the CHARMM program to add Drude oscillators and lone pairs to the final coordinates from the C36m equilibration. Recently developed patches^36^ were applied to the relevant residues to create the dianionic form of the phosphorylated amino acids. TIP3P water molecules were converted to the polarizable SWM4-NDP model.^37^ Keeping all real atoms restrained, Drude oscillators were relaxed using 1000 steps of steepest descent and 500 steps of ABNR energy minimization in CHARMM. Drude equilibration was performed in OpenMM for 1 ns using a 1 fs integration step. During the equilibration, Drude oscillators were coupled to a low-temperature relative thermostat at 1 K with a friction coefficient of 20 ps^–1^. The same restraints and constraints were applied as described for the C36m equilibration, and the nonbonded settings were the same with the exception that the van der Waals potential, not force, was switched to zero from 10 to 12 Å. The final coordinates from the Drude FF equilibration were used as the starting point for 5-μs unrestrained, unbiased NPT production simulations in OpenMM for both systems.

### Gaussian-Accelerated Molecular Dynamics Protocol

Dual-boost GaMD simulations were carried out in NAMD^22^ following the protocol previously described^38^ starting from the minimized Drude FF nonphosphorylated and phosphorylated systems. The default parameter values were used for the GaMD simulations except where specified. Each simulation consisted of 2 ns conventional MD, 50 ns GaMD equilibration, and 1000 ns GaMD production simulation. The threshold energy for the boost potential was set to lower bound, such that boosts were applied when the system potential energy dropped below the maximum potential and dihedral energies. The window for statistical averaging of the potential energies and the frequency of boost recalculation was set to 400 ps. The threshold energy and *k*_0_ were monitored over time to assess convergence of GaMD equilibration. Production runs were started using the coordinates and velocities from the last step of equilibration. The GaMD production simulations were energetically reweighted using cumulant expansion to the second order in the PyReweighting toolkit.^39^ The ϕ angles of Ser33 and Ser37, phosphorylation sites of β-catenin_17–48_, were used as collective variables (CVs) for reweighting to construct the free energy surface of each system. Convergence of the simulations was assessed by generating free energy surfaces via block reweighting every 250 ns (Figure S1).

### Metadynamics Protocol

METAD simulations^23,40^ were performed for 1000 ns starting from the equilibrated Drude FF coordinates of both systems using OpenMM and PLUMED.^41^ Two METAD simulations were performed for each system using different CVs. One of the simulations biased the ϕ angles of Ser33 and Ser37 and the second biased the distance between the Cα atoms of the first and last residues, which we will refer to as “end-to-end distance”. For both simulations, Gaussian hills with a height of 1.0 kJ/mol were deposited every 1 ps and the bias factor, γ, was set to 20. The σ value for the dihedrals was set to π/12 rad (15°) and for the end-to-end distance it was set to 0.2 Å. To assess convergence, free energy surfaces were reconstructed using the time-dependent c­(t) reweighting approach, with the corresponding CV(s) evaluated every 250 ns (Figures S2 and S4). The time series of all the CVs used are also reported (Figure S5).

### Weighted Ensemble Simulation Protocol

WE simulations were performed with the Weighted Ensemble Simulations Toolkit with Parallelization and Analysis (WESTPA) software,^24,42^ applying the Drude FF and OpenMM as recently described.^43^ We performed two different WESTPA runs with different progress coordinates. Similar to the METAD approach, one WESTPA run employed a progress coordinate along the ϕ angles of Ser33 and Ser37, for which we defined 15°-bins spanning from 0° to 360°, inclusive of end values. The other WESTPA run used the end-to-end distance, defined as in the METAD simulations, as its progress coordinate. For this progress coordinate we constructed ∼30 bins spanning from 0 Å to 70 Å. In both runs, 200 WESTPA iterations were performed with a τ of 250 ps and 5 walkers per bin starting from the equilibrated Drude coordinates of both the phosphorylated and nonphosphorylated systems. To assess convergence, we used WEDAP^44^ to generate one- and two-dimensional probability projections along the end-to-end distance and the ϕ angles of Ser33 and Ser37, respectively, every 50 iterations (Figures S3 and S4). The evolution of the CVs across iterations for each simulation is also reported (Figure S6).

### Comparison of Sampling across Methods and Phosphorylation State

Two main analyses were performed to evaluate and compare the conformational sampling obtained from each simulation method and phosphorylation state of β-catenin_17–48_: the Energy Landscape Visualization Method (ELViM)^45,46^ and *k*-means *N*-Ary Natural Initiation (NANI), as implemented in MDANCE.^47^ For these analyses, all trajectories were aligned in CHARMM using Cα atoms and a reference starting structure to ensure a consistent orientation. Trajectories were subsampled to obtain approximately 10,000 conformations per simulation to ensure balanced representation across all methods. ELViM was first applied separately for each phosphorylation state and CV, using concatenated trajectories from unbiased MD, GaMD, WESTPA, and METAD simulations, resulting in ∼40,000 conformations total per projection. Because unbiased MD and GaMD simulations were independent of CV choice, the same trajectories were used for both CV-specific projections, whereas WESTPA and METAD trajectories differed according to the CV employed. ELViM was also performed separately for each CV using concatenated trajectories from both phosphorylation states and all four simulation methods (∼80,000 conformations total), allowing direct visualization and comparison of phosphorylated and nonphosphorylated conformational ensembles within the same projected space. No reweighting was applied to configurations obtained from enhanced sampling methods prior to ELViM projection. Therefore, the distributions observed in the embedded space reflect the sampling behavior of each method rather than equilibrium populations.

The combined trajectories were also analyzed using the k-means NANI algorithm in MDANCE to cluster conformations and quantitatively assess how different simulations explored conformational space. When analyzing the combined trajectories separated by phosphorylated state, we set the number of clusters to be equal to the number of methods to facilitate direct comparison of each method’s contribution to the conformational ensemble. When analyzing the combined trajectories separated by CV but not by phosphorylation state, the Davies-Bouldin index and its derivatives were used to identify candidate cluster numbers (Figure S7). The final number of clusters was selected from these candidates by excluding solutions that merged structurally distinct conformations or resulted in an excessive number of sparsely populated clusters. Cluster structural features were characterized by extracting the 100 frames closest to each cluster centroid and analyzing the ϕ dihedral angles of the phosphorylation sites (Ser33 and Ser37) as well as calculating the helicity. Dihedral analysis was performed through MDAnalysis^48,49^ and helicity was calculated using the DSSP algorithm^50,51^ as the fraction of residues assigned α-, 3_10_-, or π-helical secondary structure.

### Comparison to NMR Observables

Every frame of the trajectories described above was used to compute ensemble-averaged NMR observables for each simulation method and phosphorylation state. These observables were calculated directly from the sampled configurations without reweighting. As such, the reported values represent ensemble averages over the sampled conformations for each method rather than reweighted equilibrium expectations. Calculations of ^3^J_HN‑Hα_ scalar couplings were performed with MDTraj using the “Bax2007” parameter set.^52,53^ Chemical shifts were calculated by using the SPARTA+ software.^54^ The calculated ^3^J_HN‑Hα_ scalar couplings and chemical shifts were compared with the experimental data reported by Megy et al.,^20^ and agreement was quantified using root-mean-square error (RMSE).

## Results and Discussion

The goal of this study was to evaluate the sampling of nonphosphorylated and phosphorylated β-catenin, as an example IDP, achieved by different enhanced sampling methods in comparison to conventional or unbiased MD simulations using the Drude FF. As stated previously, studying both states of IDPs is important given their relationship to human health and disease. Of particular interest, identifying potential transition or intermediate conformations could provide insight into what drives phosphorylation-induced conformational change. As a case study, we used a peptide derived from the N-terminal intrinsically disordered region of β-catenin, residues 17–48 (β-catenin_17–48_), given its biological relevance, size, and the existence of phosphorylation sites at Ser33 and Ser37. We selected three enhanced sampling methods that operate through distinct sampling paradigms in order to evaluate how different approaches influence exploration of conformational space: GaMD, WESTPA, and METAD. For WESTPA and METAD, we approached sampling by defining two CVs and employing them independently to report on secondary structure (Ser33 and Ser37 ϕ angles), which can change upon phosphorylation in some IDPs,^55^ including β-catenin_17–48_,^20^ and structural compactness (end-to-end distance), a property historically challenging to reproduce in MD simulations of IDPs.^56–58^ These collective variables may not fully capture all slow degrees of freedom of the system and therefore do not ensure complete convergence of the underlying free energy landscape, but instead provide a physically motivated and consistent framework for comparing how different enhanced sampling approaches explore conformational space.

### Comparison of Conformational Ensembles

The first approach used to compare sampling across methods was ELViM projections of the conformational ensembles obtained from all simulations, separated by phosphorylation state and CV definition (Figure 1). Because these projections are constructed from unweighted configurations, the distributions shown reflect method-dependent sampling rather than equilibrium conformational populations. In this approach, we were interested in evaluating how each enhanced method compared to the sampling achieved by unbiased MD. One might expect enhanced methods to both reproduce conformations observed in unbiased MD and move into new conformational space. Instead, Figure 1 shows limited overlap between the conformational ensembles generated by each method across phosphorylation state and CV definitions, indicating that no single method sampled the full conformational phase space of β-catenin_17–48_. Among the enhanced sampling approaches, GaMD sampling was the most similar to that of unbiased MD, particularly for phosphorylated β-catenin_17–48_. The extent of overlap of METAD or WESTPA sampling and unbiased MD appeared to depend more strongly on phosphorylation state than on the choice of CV. For nonphosphorylated β-catenin_17–48_, METAD sampled conformations that were largely distinct from those observed in unbiased MD, whereas greater overlap was observed for phosphorylated β-catenin_17–48_, especially when Ser33 and Ser37 ϕ angles were used as the CV. In contrast, WESTPA sampling of nonphosphorylated β-catenin_17–48_ occupied intermediate regions among those sampled by unbiased MD, GaMD, and METAD, effectively bridging these conformational ensembles. However, for phosphorylated β-catenin_17–48_, WESTPA sampling minimally overlapped with that of other methods. These trends are further illustrated in Figure S8, which shows ELViM projections grouped only by phosphorylation state.

**Figure 1:**
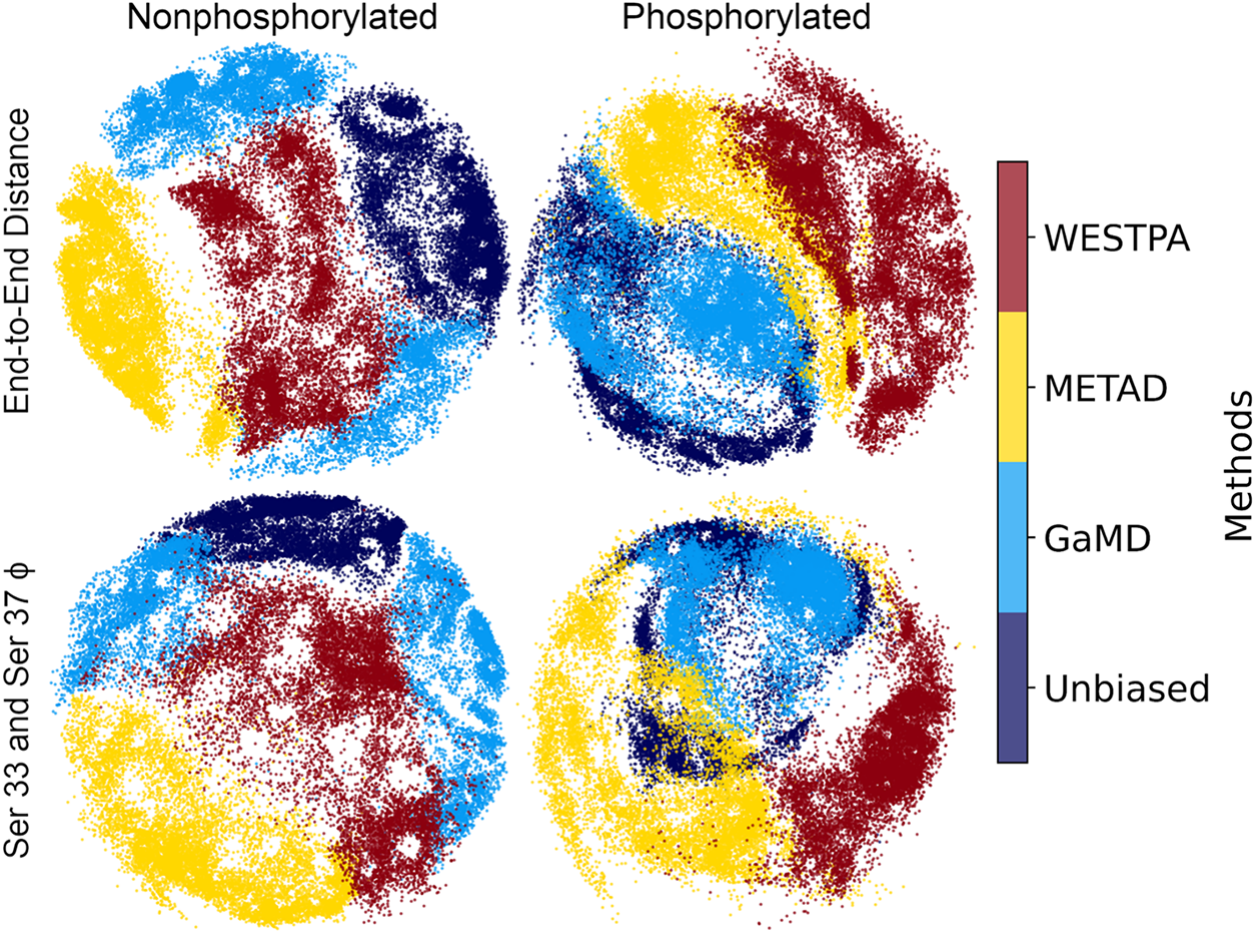
ELViM projections of the conformational ensembles of β-catenin_17–48_ obtained from each of the indicated methods, shown separately for each phosphorylation state and CV. In each panel, trajectories from all four methods were concatenated and projected together. Each dot represents a single conformation and is colored according to the simulation method, as indicated by the color bar.

To analyze these trends from a more quantitative perspective, the same concatenated trajectories were analyzed using the k-means NANI clustering algorithm in MDANCE. We set the number of clusters to be equal to the number of sampling methods (four) to facilitate direct comparison of the contribution of each method to the conformational ensemble (Figure 2). If all methods explored the same conformations, each cluster would be expected to contain approximately equal contributions from each method. Instead, unequal contributions from each method were observed across clusters. In an ideal case, enhanced sampling methods would populate all clusters sampled by unbiased MD; however, we did not observe such an outcome for all phosphorylation state and CV combinations. GaMD best achieved this coverage, as nearly every cluster populated by unbiased MD also contained GaMD conformations. METAD again exhibited a phosphorylation-state dependence, showing limited overlap with unbiased MD clusters for nonphosphorylated β-catenin_17–48_ but greater overlap for phosphorylated β-catenin_17–48_. WESTPA clustering outcomes were influenced by both CV selection and phosphorylation state. When using the Ser33 and Ser37 ϕ angles as CVs, WESTPA sampled clusters populated by unbiased MD in the nonphosphorylated state but showed less overlap in the phosphorylated state. In contrast, when using end-to-end distance as the CV, WESTPA produced good overlap with unbiased MD sampling in both phosphorylation states. Notably, METAD and WESTPA more extensively sampled clusters with little or no contribution from unbiased MD, indicating their ability to access conformational regions not observed in the unbiased simulations more frequently than GaMD.

**Figure 2:**
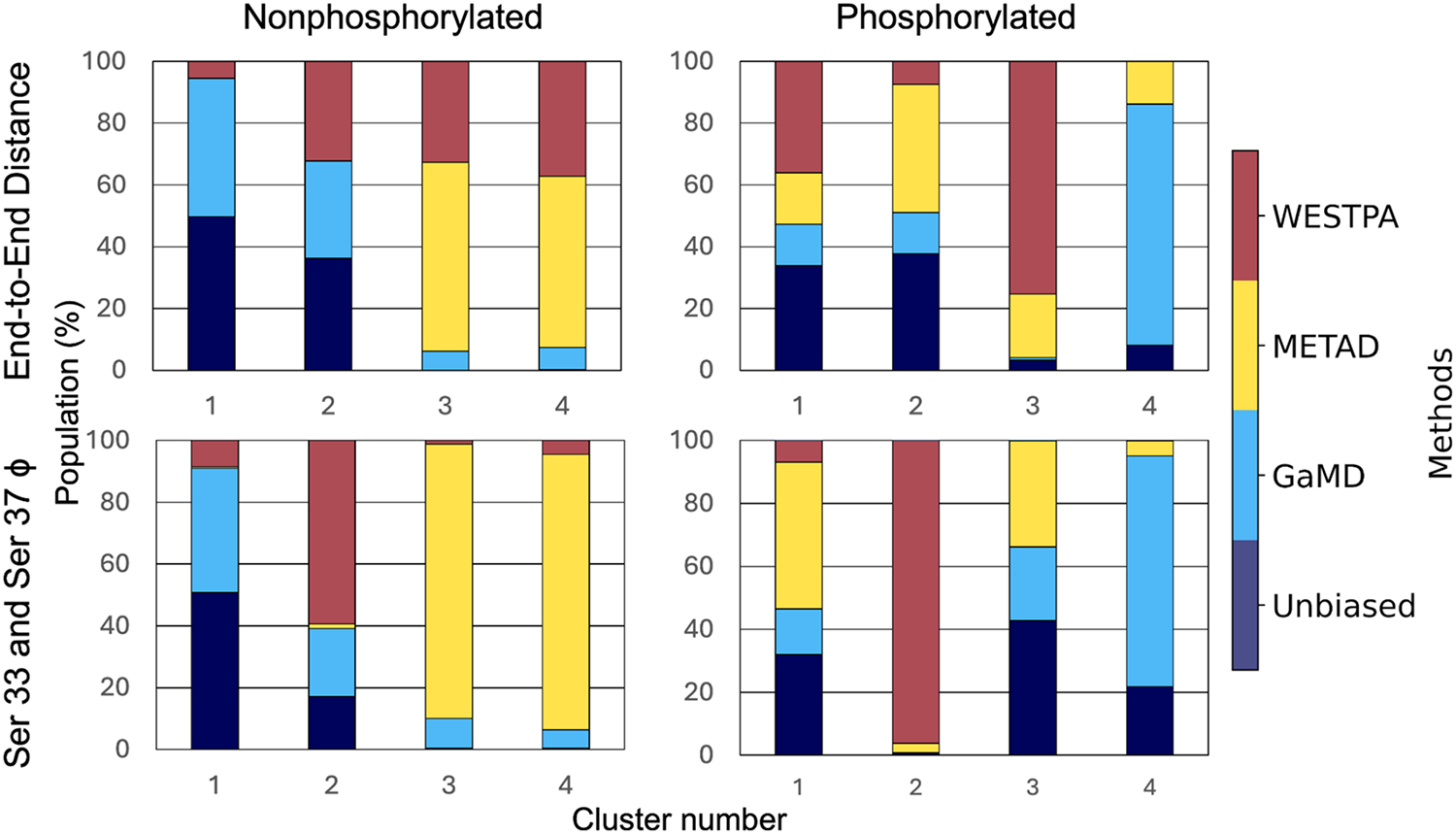
Cluster population analysis of β-catenin_17–48_ obtained using MDANCE k-means NANI, shown separately for each phosphorylation state and CV definition. In each panel, trajectories from unbiased MD, GaMD, METAD, and WESTPA were concatenated and clustered together. Stacked bar graphs show the percentage of frames contributed by each simulation method to each cluster. Colors correspond to simulation methods and follow the same color scheme used in the ELViM projections and indicated by the color bar.

The different sampling behavior observed in the ELViM projections and clustering analysis for GaMD, METAD, and WESTPA could be rationalized by their distinct enhanced sampling strategies. GaMD applies a nonadaptive boost potential to the system potential energy to smooth the underlying energy landscape, whereas METAD employs an adaptive biasing potential by depositing bias along predefined CVs and WESTPA uses adaptive sampling to redistribute simulation effort across regions of conformational space.^17^ In both METAD and WESTPA, the sampling strategy responds dynamically to the regions of conformational space already explored, as determined by the chosen CVs. In contrast, GaMD does not explicitly guide sampling along particular structural coordinates but instead facilitates barrier crossing through modification of the potential energy surface. IDPs are known to sample along free-energy landscapes containing multiple local minima separated by relatively small barriers,^59^ therefore, the challenge in sampling these systems may lie less in overcoming large energetic barriers and more in efficiently exploring diverse regions of conformational space. Methods that adaptively direct sampling toward underexplored regions, such as METAD and WESTPA, may therefore be more effective at expanding the accessible conformational landscape than approaches like GaMD that primarily enhance barrier crossing. This behavior is consistent with the observation that both methods more frequently sampled clusters with little or no contribution from unbiased MD, whereas GaMD sampling largely overlapped with conformations already visited in unbiased simulations. Taken together, these results indicate that different enhanced sampling strategies provide complementary views of the β-catenin_17–48_ conformational landscape rather than converging on a single ensemble. For intrinsically disordered systems, this outcome suggests that capturing functionally relevant conformations may require combining sampling methods rather than relying on a single method or carefully selecting methods based on the specific sampling challenges of the system to adequately capture the structural heterogeneity underlying IDP function and regulation.

### Impact of Phosphorylation on the Ensemble of β-Catenin_17–48_

As previously mentioned, we were also interested in whether any of the sampling methods could identify potential transition or intermediate conformations that might provide insight into what drives phosphorylation-induced conformational change. To assess this, and to characterize the effect of phosphorylation on the conformational space of β-catenin_17–48_, we used ELViM to generate projections of the conformational space sampled across all simulations, separated only by CV definition (Figures 3 and 4). The projections reveal that the conformational spaces sampled by the two phosphorylation states are largely distinct. In general, the region occupied by phosphorylated β-catenin_17–48_ appears more compact and localized than that sampled by the nonphosphorylated form. The two phosphorylation states are completely separated when end-to-end distance is used as the CV (Figure 3), whereas partial overlap is observed when the Ser33 and Ser37 ϕ angles are used (Figure 4). As expected, because the GaMD input data are identical in both projections, this overlap is possible thanks to WESTPA and METAD. These two methods are also the only ones that approach the boundary between the phosphorylation states in the end-to-end projection. Overall, this analysis further illustrates that GaMD primarily enriches sampling near conformations already visited by unbiased MD, whereas WESTPA and METAD more readily explore new regions of conformational space.

**Figure 3:**
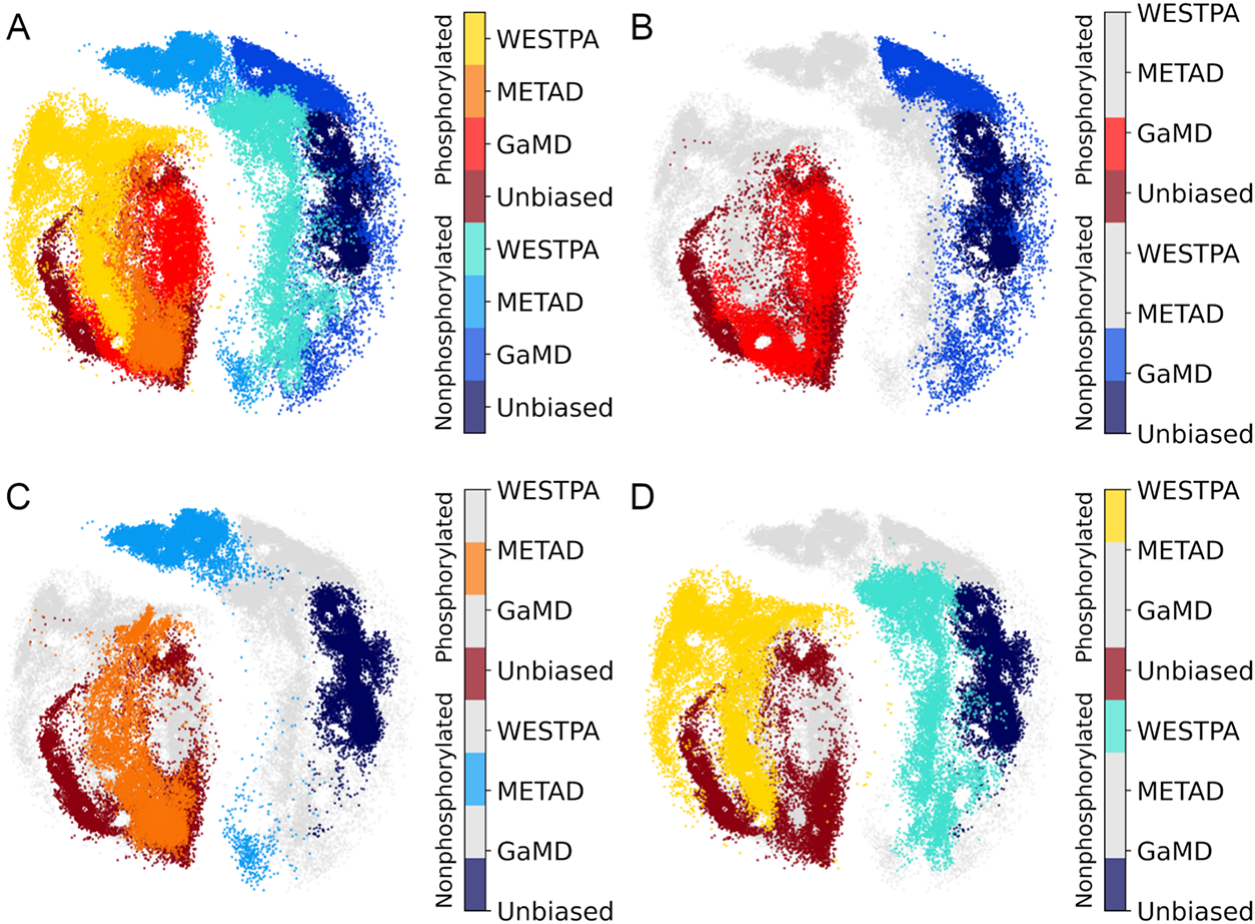
ELViM projections of the conformational ensembles of nonphosphorylated and phosphorylated β-catenin_17–48_ obtained from the indicated methods using end-to-end distance as the CV for WESTPA and METAD. Trajectories from all eight trajectories were concatenated and projected together with each dot representing a single conformation. (A) Projection including conformations from all simulation methods and phosphorylation states, colored according to method and phosphorylation state as indicated by the color bar. (B–D) Comparisons between unbiased simulations and (B) GaMD, (C) METAD, or (D) WESTPA. Conformations from the highlighted method and unbiased simulations are shown in color, while all other conformations are shown in gray to facilitate comparison within the same projection.

**Figure 4:**
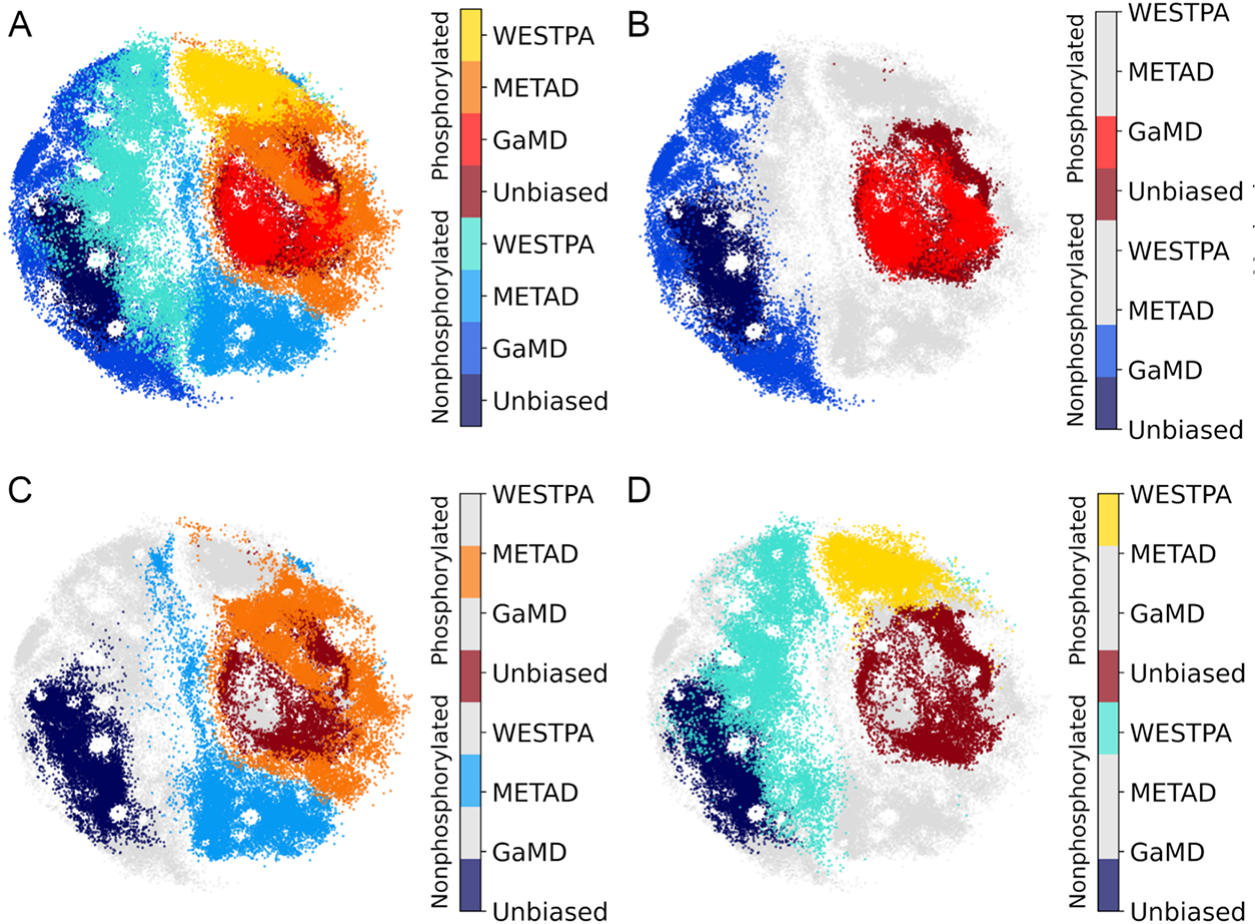
ELViM projections of the conformational ensembles of nonphosphorylated and phosphorylated β-catenin_17–48_ obtained from the indicated methods using Ser33 and Ser37 ϕ angles as CVs for WESTPA and METAD. Trajectories from all eight trajectories were concatenated and projected together with each dot representing a single conformation. (A) Projection including conformations from all simulation methods and phosphorylation states, colored according to method and phosphorylation state as indicated by the color bar. (B–D) Comparisons between unbiased simulations and (B) GaMD, (C) METAD, or (D) WESTPA. Conformations from the highlighted method and unbiased simulations are shown in color, while all other conformations are shown in gray to facilitate comparison within the same projection.

We next applied k-means clustering using NANI on the concatenated trajectories grouped by CV but not by phosphorylation state. In this case, the Davies-Bouldin index was used to identify candidate cluster numbers (Figure S7), resulting in an optimal solution of 11 clusters for both CV definitions (Figure 5). The clustering results further support the trends observed in the ELViM projections. When the end-to-end distance was used as the CV, none of the clusters contained contributions from both nonphosphorylated and phosphorylated simulations, indicating that the conformational ensembles of the two phosphorylation states remain well separated under this CV definition. In contrast, when the Ser33 and Ser37 ϕ angles were used as CVs, four of the 11 clusters contained at least 5% contributions from both phosphorylation states (clusters 2, 5, 6, and 10). These mixed clusters may correspond to conformational regions shared between the two states and therefore represent potential intermediate or transition-like states along the phosphorylation-dependent conformational shift.

**Figure 5:**
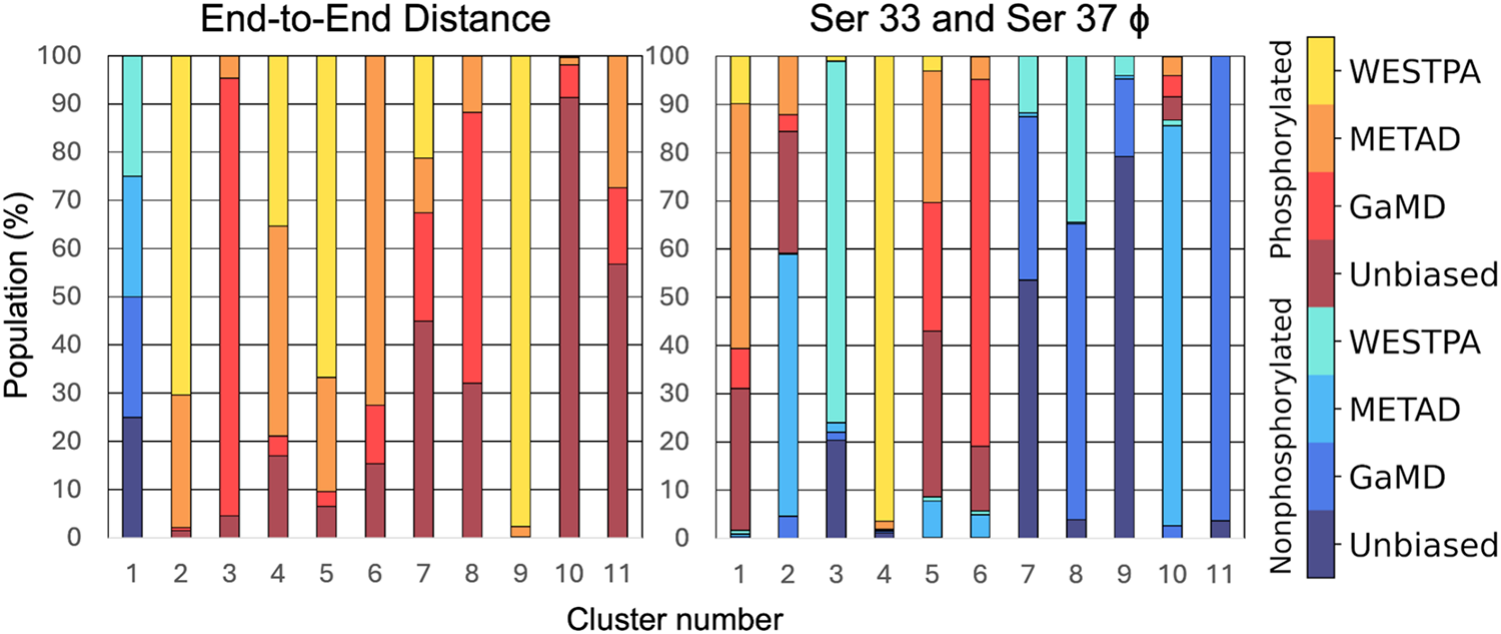
Cluster population analysis of nonphosphorylated and phosphorylated β-catenin_17–48_ obtained using MDANCE k-means NANI, shown separately for each CV definition. In each panel, trajectories from all eight simulations were concatenated and clustered together. Stacked bar graphs show the percentage of frames contributed by each simulation method of each phosphorylation state to each cluster. Colors follow the same color scheme used in the ELViM projections and are indicated by the color bar.

Examination of the simulation contributions indicates that these mixed clusters arise primarily from conformations sampled in the METAD simulations of the nonphosphorylated system, together with structures from unbiased MD, GaMD, and METAD in the phosphorylated system. Because the unbiased MD and GaMD trajectories are identical in both clustering analyses, the emergence of mixed clusters only in the ϕ-angle clustering indicates that sampling along these CVs enables exploration of conformational regions connecting the two phosphorylation states, with METAD contributing most strongly to this cross-sampling. Consistent with the ELViM projections, conformations from these simulations appear near the boundary between the conformational regions associated with each phosphorylation state (Figure 4). Some clusters (1, 3, and 4) contain minor contributions (<5%) from the opposite phosphorylation state. These likely reflect the limited cross-sampling visible in the ELViM projections, where a small number of data points, primarily from METAD and WESTPA, appear in regions predominantly associated with the opposite phosphorylation state.

To further characterize these clusters, we compared the cluster center structures with the starting NMR structures for both phosphorylation states (Figure 6) and analyzed the Ser33 and Ser37 ϕ angles as well as the helicity of the 100 frames closest to each cluster centroid (Figure S9). Comparison of the cluster centers with the NMR structures shows that clusters primarily populated by phosphorylated simulations, such as clusters 5 and 6, closely resemble the phosphorylated β-catenin_17–48_ NMR structure, which contains an N-terminal α-helix, demonstrating that the simulations capture the experimentally observed phosphorylation-induced structural transition. In contrast, clusters primarily populated by nonphosphorylated simulations, such as clusters 7 and 11, more closely resemble the nonphosphorylated β-catenin_17–48_ NMR structure, which adopts a compact and disordered conformation, further reinforcing the agreement between the simulated ensembles and experimental observations. However, inspection of the representative structures alone does not clearly indicate whether intermediate conformations along the phosphorylation-induced structural change were sampled.

**Figure 6:**
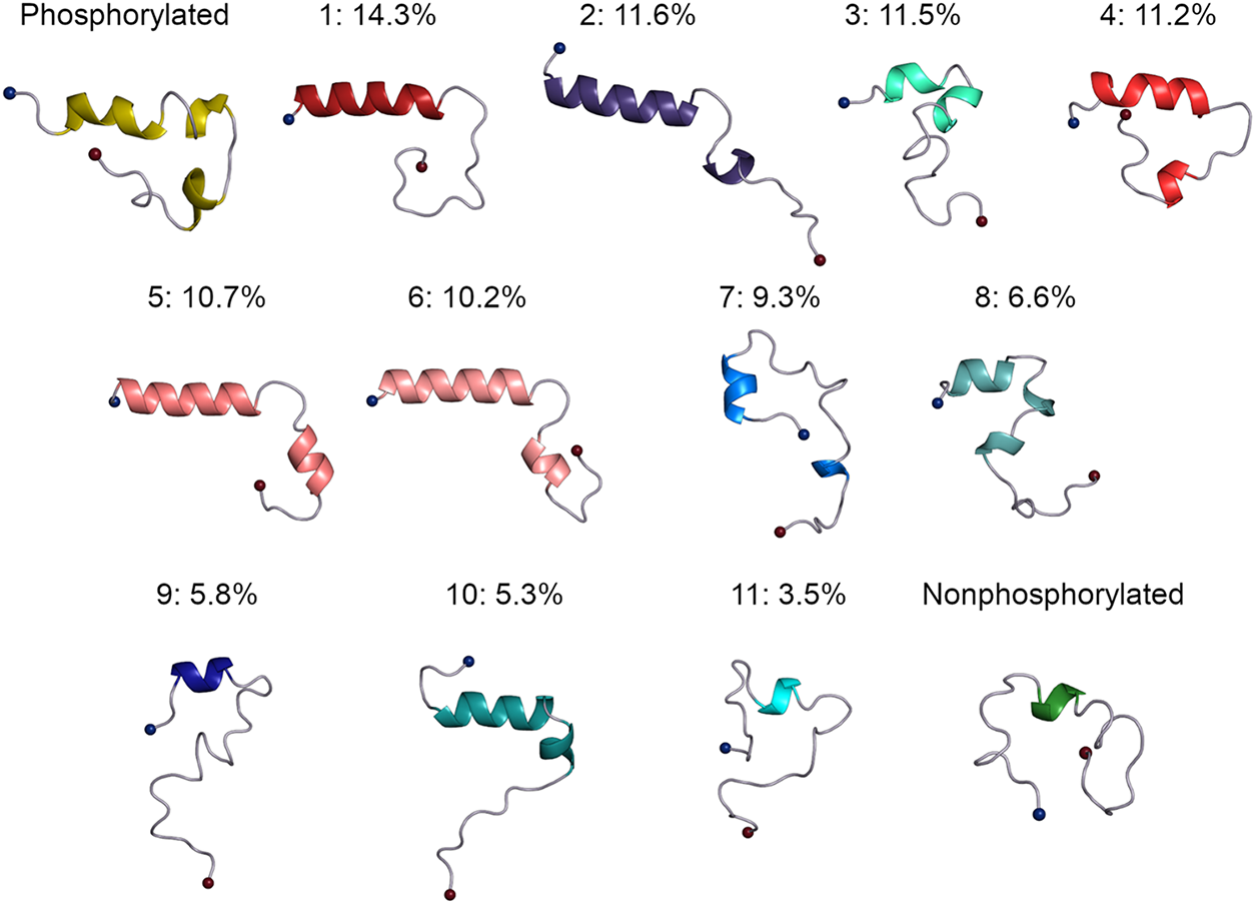
Cluster center structures from k-means clustering using NANI on the concatenated trajectories with the Ser33 and Ser37 ϕ angles as CVs. Percentages indicate the fraction of the ∼80,000 total frames assigned to each cluster. Structures are shown in cartoon representation and colored according to the predominant phosphorylation state contributing to each cluster: shades of red indicate clusters primarily populated by phosphorylated simulations, shades of blue indicate clusters primarily populated by nonphosphorylated simulations, and purple is a cluster with substantial contributions from both phosphorylation states. The starting structures for the nonphosphorylated and phosphorylated systems are shown in green and gold, respectively. The N- and C-termini are shown as spheres and colored blue and red, respectively.

Analysis of the average Ser33 and Ser37 ϕ angles (Figure S9A) shows that most clusters sample values near the energy minima observed for both phosphorylation states in the free-energy landscapes obtained from the enhanced sampling simulations (Figures S1–S3), around −60° for both residues. Clusters 3 and 8, which are primarily populated by nonphosphorylated simulations, appear to sample states primarily accessible in the nonphosphorylated simulations. Interestingly, cluster 4, although predominantly populated by phosphorylated WESTPA simulations, also samples this region. Examination of the average helicity (Figure S9B) reveals a clearer separation between clusters primarily populated by phosphorylated simulations and those dominated by nonphosphorylated simulations. Clusters 3 and 4 again display behavior distinct from the other clusters and show greater variability in helicity. In addition, cluster 10 exhibits helicity values characteristic of both phosphorylation states despite being primarily populated by nonphosphorylated METAD simulations, suggesting that nonphosphorylated ensembles can transiently access conformations resembling the phosphorylated state. Interestingly, clusters displaying these intermediate characteristics tend to arise from simulations employing METAD (cluster 10) or WESTPA (clusters 3 and 4), rather than from cluster 2, which initially appeared promising due to its mixed contributions (60:40) from nonphosphorylated and phosphorylated simulations. This observation suggests that adaptive methods can capture rare or transient conformations that may be functionally relevant, even when they are not heavily populated across both phosphorylation states. Taken together, these results suggest that certain clusters sampled by METAD and WESTPA capture conformational features intermediate between the nonphosphorylated and phosphorylated states, providing evidence that these methods can identify transition-like conformations consistent with experimentally observed structural differences, thereby supporting their ability to capture biologically relevant states.

The differences observed between the two CV definitions further highlight how the choice of CV influences the ability to detect such intermediate conformations. While both end-to-end distance and Ser33/Ser37 ϕ angles enabled broad sampling of each phosphorylation state (Figure 1), only the ϕ angle CVs led to cross-sampling between the two states. This behavior may arise because perturbations of the ϕ angles more directly mimic the structural effects of phosphorylation on local backbone conformations. Changes in these local structural features can propagate to influence the global conformation of the peptide. Consistent with this interpretation, the free-energy landscapes obtained from METAD and WESTPA simulations show a more pronounced shift in the preferred ϕ angle values upon phosphorylation than in the end-to-end distance distributions (Figures S2–S4). We note, however, that the choice of CVs can strongly influence sampling outcomes. In particular, end-to-end distance is a known degenerate CV, such that important slow degrees of freedom may not be fully captured when biasing along this coordinate. More generally, identifying optimal CVs for intrinsically disordered proteins remains a nontrivial challenge. Nonetheless, our observations suggest that variations in the Ser33 and Ser37 ϕ angles may serve as a more sensitive indicator of phosphorylation-induced conformational change than global measures such as end-to-end distance. Therefore, local structural descriptors linked to modification sites may be critical for uncovering the conformational transitions of IDPs and appropriately chosen sampling strategies may be essential for capturing these biologically relevant states.

### Validation of Simulations with NMR Observables

Thus, far, we have discussed the performance of each method with respect to conformational sampling and the ability to capture phosphorylation-induced structural changes. However, it is also important to evaluate both the precision and accuracy of the simulations. With respect to precision, the free-energy landscapes obtained from the different sampling methods in this study show broadly similar features for both CV definitions, indicating consistent characterization of the underlying conformational preferences across independent simulations. To assess accuracy, we calculated NMR observables from the simulated ensembles and compared them with experimental measurements reported by Megy et al.^20^ For the ^3^J_HN‑Hα_ couplings, we observed good agreement with experiments across all methods for the nonphosphorylated simulations. In contrast, the calculated values for the phosphorylated system were generally lower than the experimental couplings, particularly for residues that form the N-terminal α-helix (Figure 7). A similar outcome is observed for the Cα chemical shifts: agreement with experiment is strong for the nonphosphorylated simulations, whereas the calculated shifts were systematically higher in the phosphorylated system for N-terminal residues. In comparison, the Cβ chemical shifts showed relatively good agreement with experiment for both phosphorylation states. These trends are reflected in the corresponding RMSE values relative to the experimental data (Tables S1 and S2). Overall, the agreement with experiment is broadly similar across the different sampling methods, although WESTPA simulations consistently yielded slightly better agreement (quantified via lower RMSE values) than the other approaches. These results indicate that, despite differences in sampling behavior, all methods produced conformational ensembles that are broadly consistent with available NMR observables. Therefore, even for a challenging system such as β-catenin_17–48_, all sampling methods were able to accurately capture the ensemble properties of this IDP.

**Figure 7:**
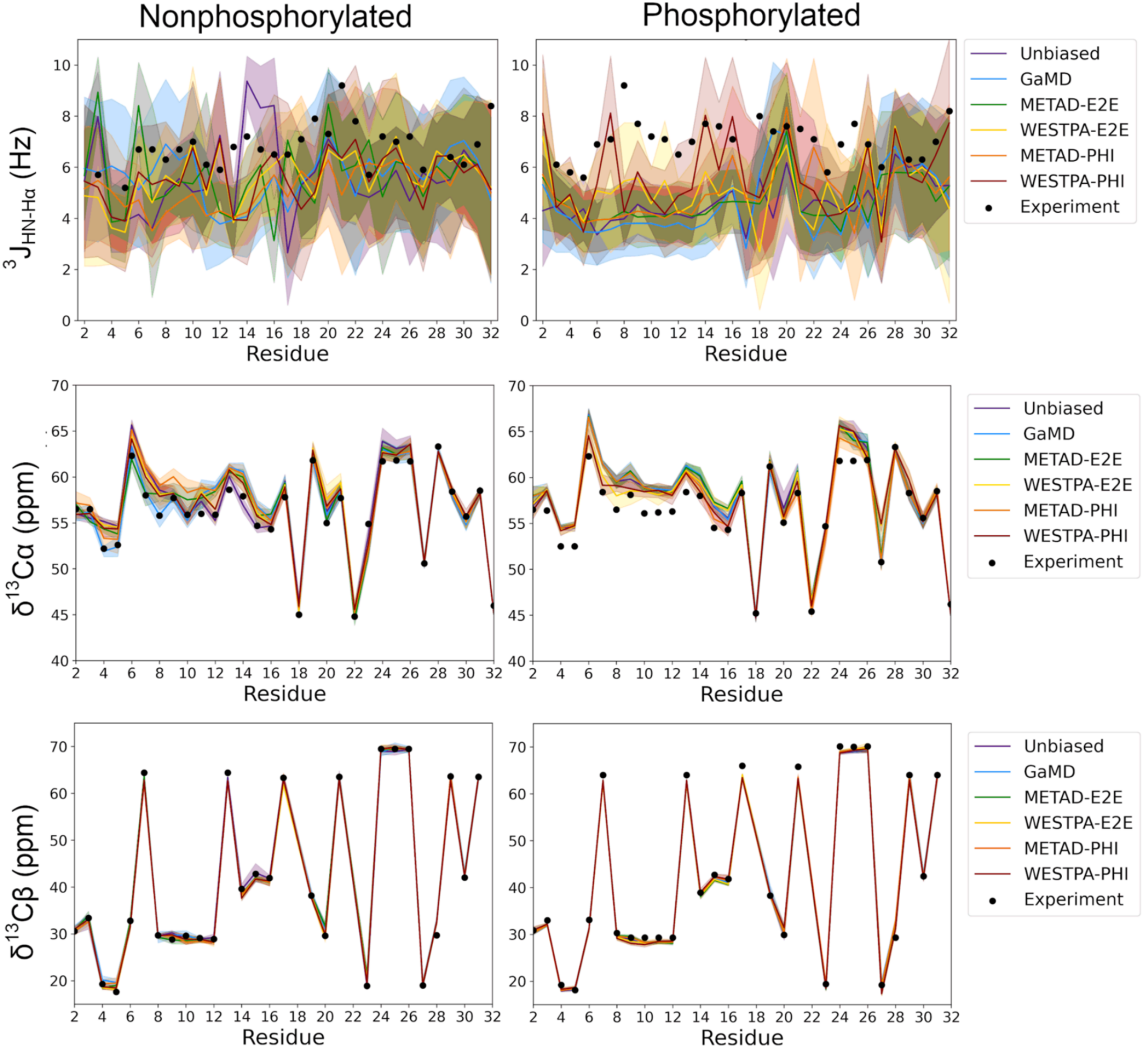
Comparison of simulated and experimental NMR observables for β-catenin_17–48_. Simulation results from the six methods are shown as colored lines, while experimental values are shown as black dots, as indicated in the legend.

## Conclusions

In this work, we evaluated how different enhanced sampling strategies influence exploration of the conformational landscape of the intrinsically disordered β-catenin_17–48_ peptide in both its nonphosphorylated and phosphorylated states. By combining dimensionality reduction, clustering analysis, and comparison with experimental NMR observables, we assessed the ability of Gaussian accelerated molecular dynamics (GaMD), well-tempered metadynamics (METAD), and weighted ensemble simulations (WESTPA) to sample relevant conformational ensembles.

Overall, the enhanced sampling methods explored complementary regions of conformational space rather than converging to a single shared ensemble. GaMD sampling largely overlapped with conformations observed in conventional molecular dynamics simulations, indicating that the method primarily enriches sampling near already visited states. In contrast, METAD and WESTPA more frequently accessed conformational regions not sampled by unbiased MD, highlighting the ability of adaptive biasing and adaptive sampling approaches to promote exploration of under-sampled regions of the landscape.

Analysis of the combined conformational ensembles further suggested the presence of intermediate conformations connecting the nonphosphorylated and phosphorylated states of β-catenin_17–48_. These intermediate-like states were most prominently sampled in simulations employing METAD and WESTPA, suggesting that adaptive strategies may be particularly well suited for identifying transitional regions of IDP conformational landscapes. Additionally, the choice of collective variable was found to strongly influence sampling behavior: biasing the Ser33 and Ser37 ϕ dihedral angles enabled cross-sampling between phosphorylation states, whereas the global end-to-end distance primarily separated the two ensembles. This observation indicates that CVs directly associated with local structural perturbations induced by post-translational modification may provide more sensitive descriptors of phosphorylation-dependent conformational change.

Finally, comparison of simulated ensembles with experimental NMR observables showed broadly similar levels of agreement across the different sampling methods, suggesting that each approach produces conformational ensembles consistent with available experimental data despite differences in sampling behavior. These results not only highlight the capabilities of enhanced sampling methods to interrogate IDP conformational landscapes but also provide insights into the molecular mechanisms by which phosphorylation may modulate IDP structure, and therefore function. Future work could extend this analysis by exploring alternative collective variables and higher-dimensional CVs, evaluating additional enhanced sampling strategies, including parallel biasing approaches within metadynamics, and examining whether similar trends hold across a broader set of intrinsically disordered proteins.

## Supplementary Material



## Notes

GaMD two-dimensional free energy surfaces over time for both systems; WESTPA and METAD free energy profiles for the simulations that end-to-end distanceas progress coordinate and collective variable; RMSE between simulated and experimental NMR observables for each of the six simulation methods of the nonphosphorylated β-catenin_17–48_ system (PDF)

## §

Department of Biochemistry Federal University of Viçosa Avenida P. H. Rolfs, s/n Campus UFV, Viçosa, MG 36570-900, Brazil
