# Supplementary material for: Comparing Enhanced Sampling Methods in Exploring the Conformational Space of β‑Catenin17–48
Source: J Phys Chem B. 2026 Jun 24;130(29):7323–33. doi: 10.1021/acs.jpcb.6c02031 (PMC13403295; doi:10.1021/acs.jpcb.6c02031)
Supplement: Supplementary file 1 [file jp6c02031_si_001.pdf]

SUPPORTING INFORMATION

Comparing Enhanced Sampling Methods in Exploring the Conformational Space of β-Catenin$_{17\text{-}48}$

Laura I. Gil Pineda,[1] Marcelo D. Polêto,[1,†] Haley M. Michel,[1] Ashley M. Goodberlet,[1] and Justin A. Lemkul[1,2*]

[1] Department of Biochemistry
[2] Center for Drug Discovery
Virginia Tech, Blacksburg, VA 24061

*Corresponding Author
Email: jalemkul@vt.edu
Address: 111 Engel Hall
340 West Campus Dr.
Blacksburg, VA 24061
Phone: (540) 231-3129

† Current Address and Affiliation
MDP: Department of Biochemistry
Federal University of Viçosa
Avenida P. H. Rolfs, s/n
Campus UFV, Viçosa - MG, 36570-900, Brazil

SUPPORTING FIGURES

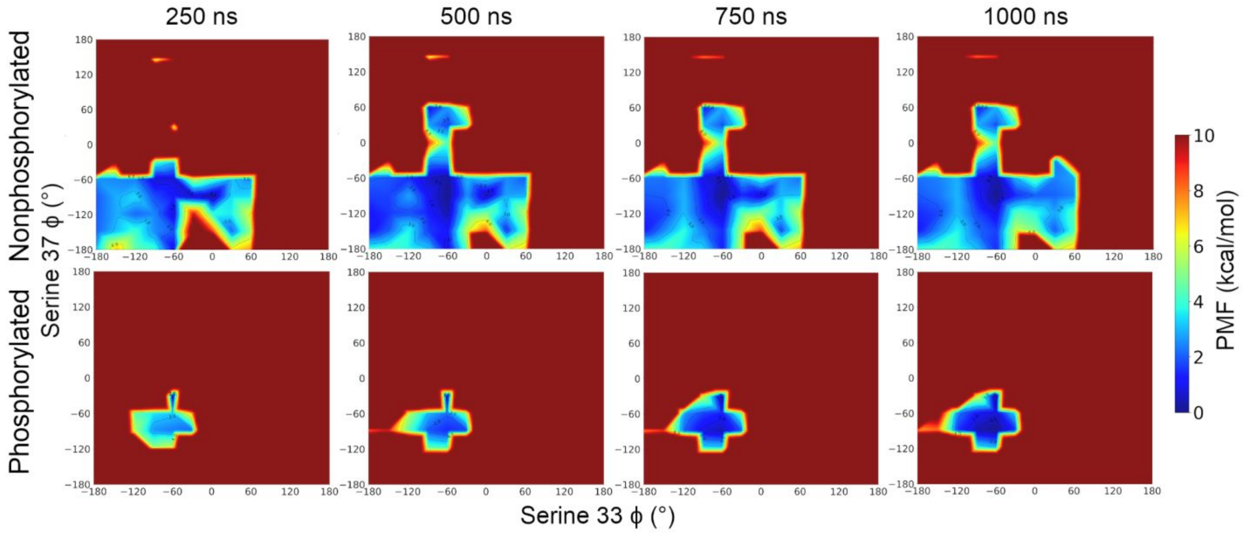

Figure S1. GaMD two-dimensional free energy surfaces over time for both systems. Data were pooled and reweighted every 250 ns using cumulant expansion to the second order.

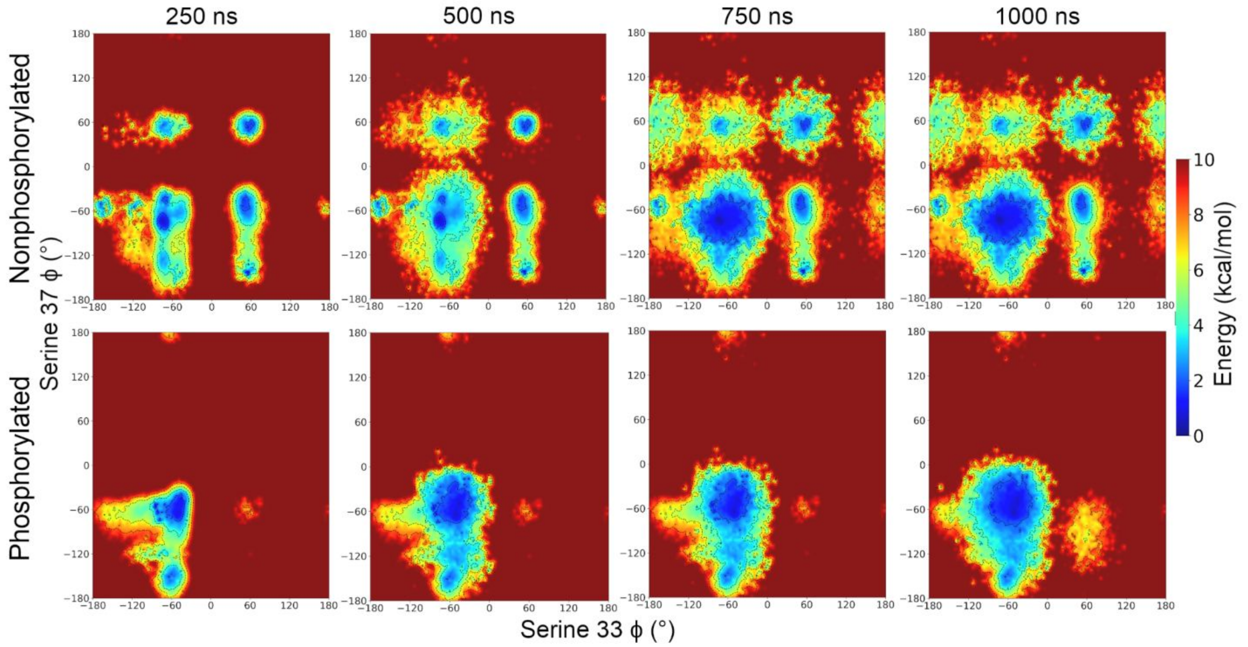

Figure S2. METAD two-dimensional free energy surfaces over time for simulations that biased the ϕ angles of Ser33 and Ser37.

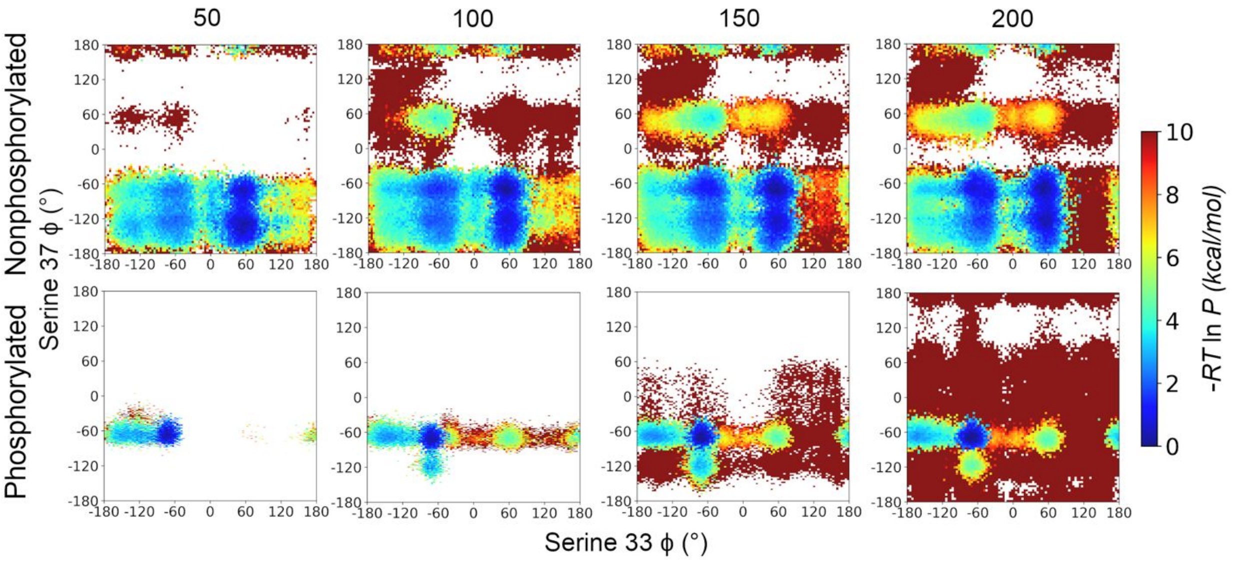

Figure S3. WESTPA two-dimensional projections for the simulations that used ϕ angles of Ser33 and Ser37 as progress coordinates, calculated every 50 iterations for both systems.

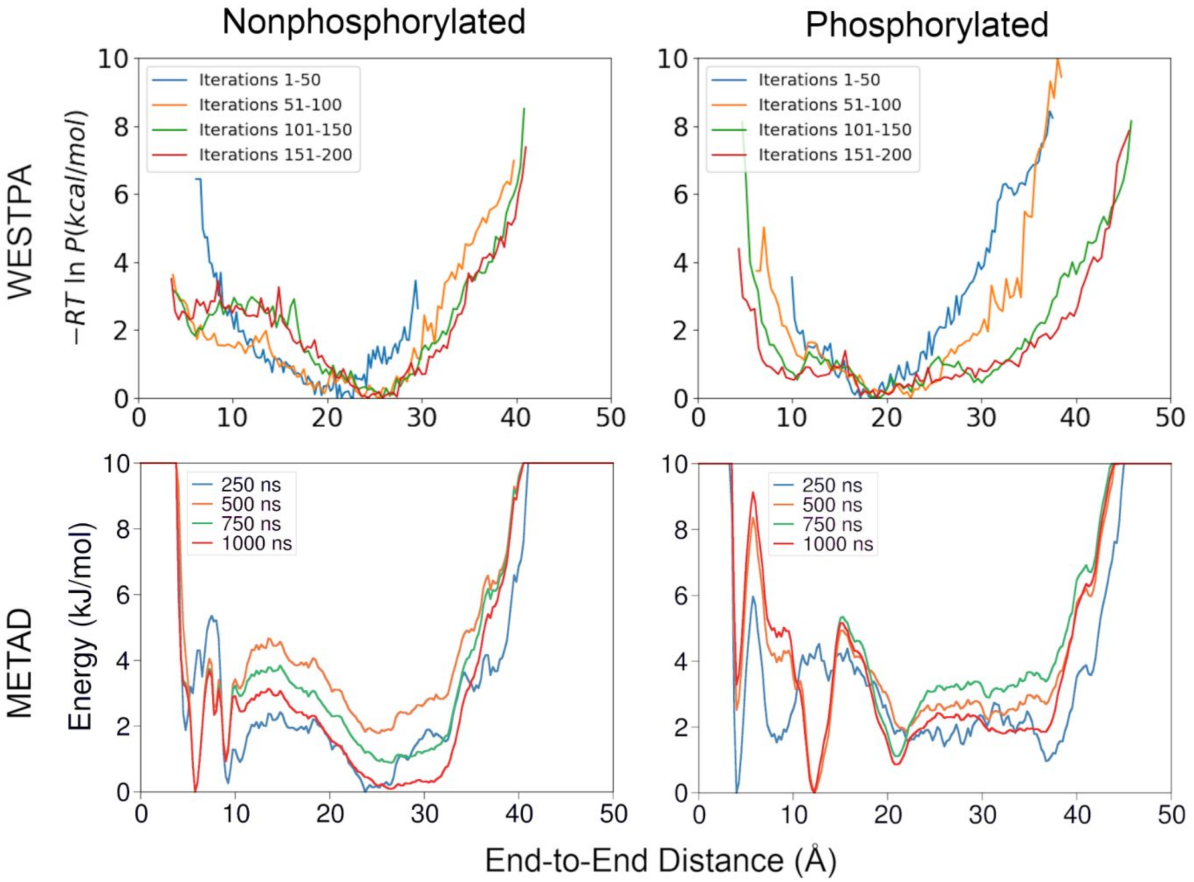

Figure S4. WESTPA and METAD free energy profiles for the simulations that end-to-end distance as progress coordinate and collective variable, respectively. WESTPA profiles were calculated every 50 iterations and METAD every 250 ns, for both systems.

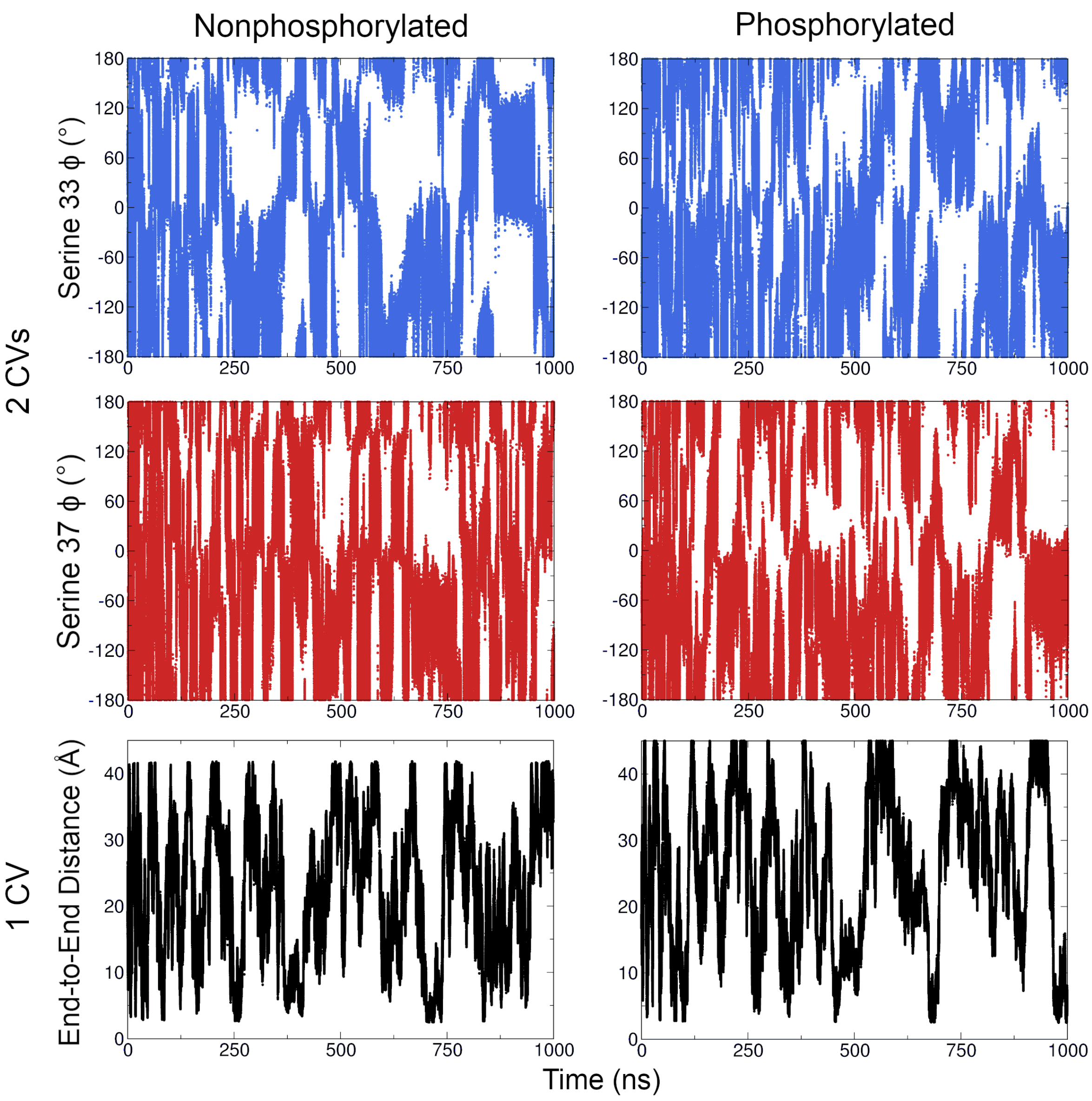

Figure S5. Time series of the collective variables used in METAD simulations of nonphosphorylated (left column) and phosphorylated (right column) β-catenin$_{17–48}$. The top and middle rows show the Ser33 and Ser37 ϕ dihedral angles, respectively, from simulations in which these dihedral angles were used as collective variables. The bottom row shows the end-to-end distance from simulations in which end-to-end distance was used as the collective variable.

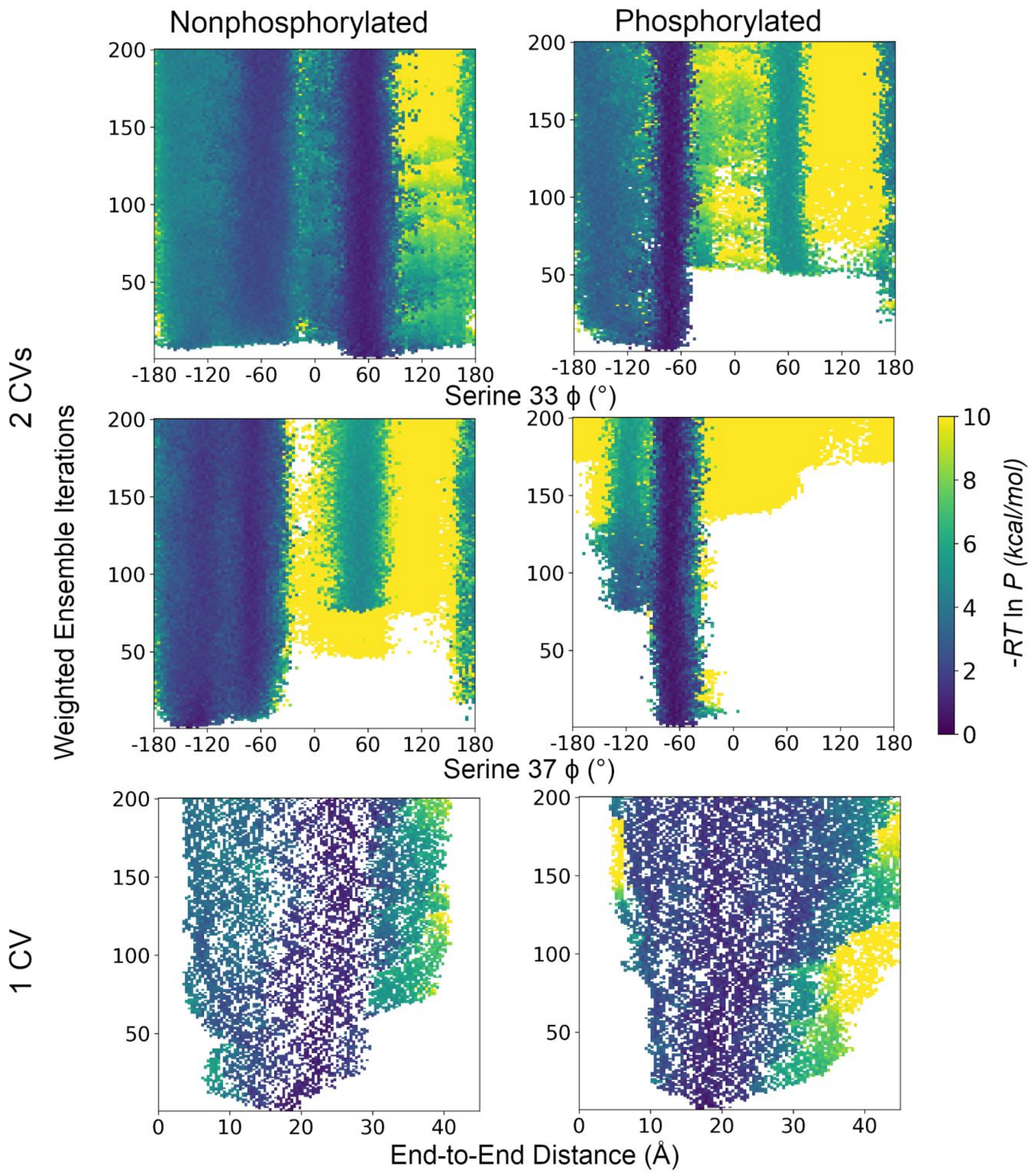

Figure S6. Evolution of the collective variables sampled during WESTPA simulations of nonphosphorylated (left column) and phosphorylated (right column) β-catenin$_{17–48}$. The top and middle rows show sampling of the Ser33 and Ser37 ϕ dihedral angles, respectively, for simulations employing the dihedral angles as collective variables. The bottom row shows sampling of the end-to-end distance for simulations employing the end-to-end distance collective variable. Plots are shown as a function of WESTPA iteration and illustrate the progressive exploration of collective variable space during the simulations.

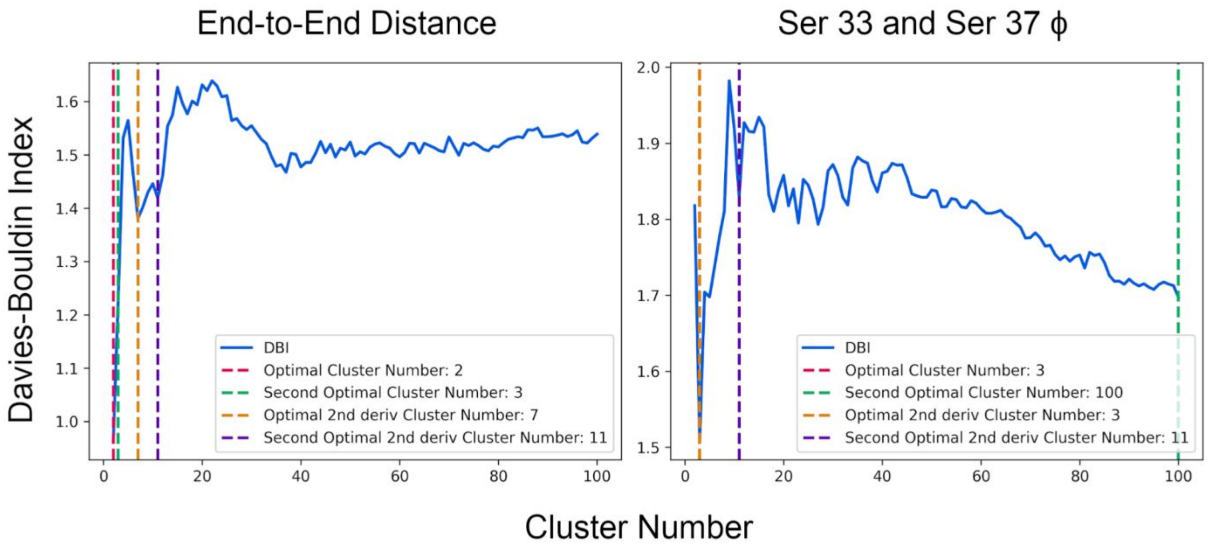

Figure S7. Davies-Bouldin index as a function of cluster number used to determine the optimal number of clusters for the k-means NANI analysis. Trajectories from all simulations were concatenated and analyzed separately for each CV definition.

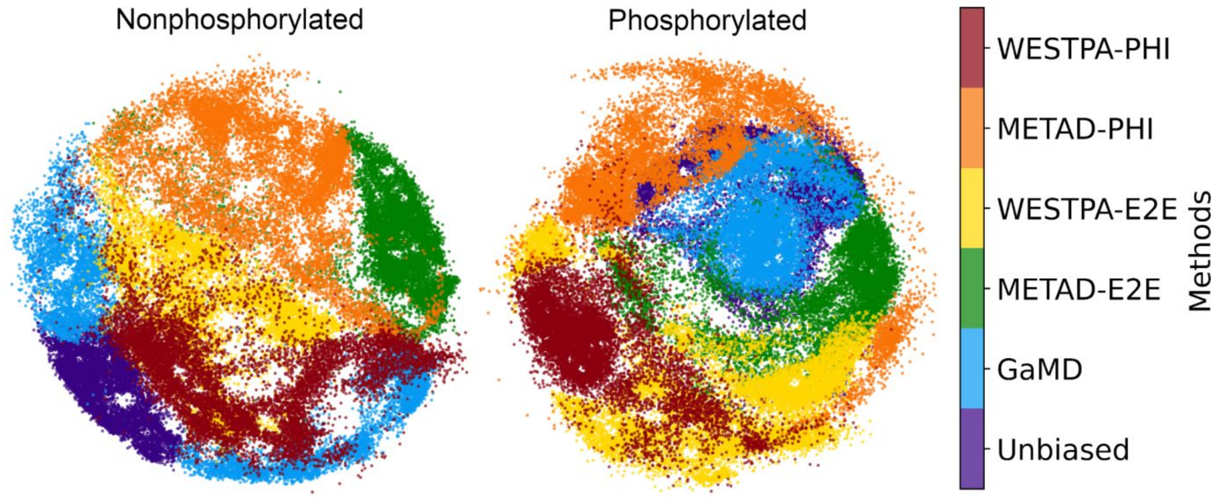

Figure S8. ELViM projections of the conformational ensembles of β-catenin$_{17\text{-}48}$ obtained from unbiased MD, GaMD, and both CV definitions used in METAD, AND WESTPA, shown separately for each phosphorylation state. In each panel, trajectories from all six approaches were concatenated and projected together. E2E refers to the end-to-end distance CV definition and PHI refers to when methods biased along the Ser33 and Ser37 ϕ angles. Each dot represents a single conformation and is colored according to the simulation method, as indicated by the color bar.

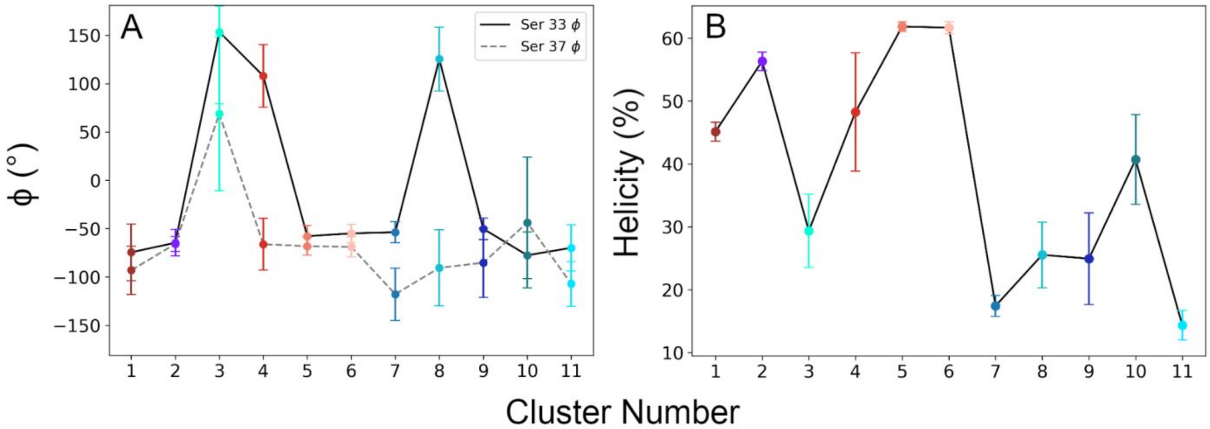

Figure S9. Structural characterization of clusters obtained from k-means clustering using NANI on the concatenated trajectories with the Ser33 and Ser37 ϕ angles as CVs.(A) Average ϕ dihedral angles of Ser33 and Ser37 as a function of cluster number. (B) Average helicity (%) as a function of cluster number. Error bars represent the standard deviation. Cluster points are colored according to the predominant phosphorylation state contributing to each cluster: shades of red indicate clusters primarily populated by phosphorylated simulations (clusters 1, 4, 5, and 6), shades of blue indicate clusters primarily populated by nonphosphorylated simulations (clusters 3, 7, 8, 9, 10, and 11), and cluster 2 is shown in purple as it contains substantial contributions from both phosphorylation states.

SUPPORTING TABLES

Table S1. RMSE between simulated and experimental NMR observables for each of the six simulation methods of the nonphosphorylated β-catenin$_{17\text{-}48}$ system. E2E refers to the end-to-end distance CV definition and PHI refers to when methods biased along the Ser33 and Ser37 ϕ angles.

	$^3J_{HN\text{-}H\alpha}$ RMSE (Hz)	δ^{13}Cα RMSE (ppm)	δ^{13}Cβ RMSE (ppm)
Unbiased	2.0423	1.4647	0.9052
GaMD	1.7345	1.2743	1.0200
METAD-E2E	1.9272	1.3576	1.0521
WESTPA-E2E	1.5562	1.4496	0.9970
METAD-PHI	1.9414	1.6844	1.0575
WESTPA-PHI	1.6083	1.2879	0.9547

Table S2. RMSE between simulated and experimental NMR observables for each of the six simulation methods of the phosphorylated β-catenin$_{17\text{-}48}$ system. E2E refers to the end-to-end distance CV definition and PHI refers to when methods biased along the Ser33 and Ser37 ϕ angles.

	$^3J_{HN\text{-}H\alpha}$ RMSE (Hz)	δ^{13}Cα RMSE (ppm)	δ^{13}Cβ RMSE (ppm)
Unbiased	2.9929	2.0229	1.2486
GaMD	3.2082	2.0838	1.2916
METAD-E2E	2.8965	2.0909	1.2758
WESTPA-E2E	2.6392	1.8173	1.2092
METAD-PHI	2.6843	1.7436	1.2114
WESTPA-PHI	2.2808	1.7360	1.2025
